# Convergent increases in serotonin 1B receptor binding following ketamine and electroconvulsive therapy: a multi-centre bayesian re-analysis of PET data

**DOI:** 10.1038/s41380-026-03704-2

**Published:** 2026-07-08

**Authors:** Granville J. Matheson, Johan Lundberg, Martin Gärde, Emma R. Veldman, Amane Tateno, Carl-Johan Ekman, Ryosuke Arakawa, Gitte M. Knudsen, Yoshiro Okubo, R. Todd Ogden, Mikael Tiger

**Affiliations:** 1 Department of Clinical Neuroscience, Center for Psychiatry Research, Karolinska Institutet and Stockholm County Council, Stockholm, SE-171 64 Sweden; 2 Department of Psychiatry, Columbia University, New York,, 10032 NY USA; 3 Department of Biostatistics, Columbia University Mailman School of Public Health, New York, 10032 NY USA; 4 Molecular Imaging and Neuropathology Division, New York State Psychiatric Institute, New York, 10032 NY USA; 5 Department of Neuropsychiatry, Graduate School of Medicine, Nippon Medical School, Tokyo, Japan; 6 Department of Pharmacology, Graduate School of Medicine, Nippon Medical School, Tokyo, Japan; 7 Neurobiology Research Unit, Copenhagen University Hospital Rigshospitalet, Copenhagen, Denmark; 8 Department of Clinical Medicine, Faculty of Health and Medicine Sciences, University of Copenhagen, Copenhagen, Denmark

**Keywords:** Neuroscience, Depression

## Abstract

The serotonin 1B receptor can be studied in vivo with PET using [^11^C]AZ10419369, and has been linked to both the pathophysiology and treatment of major depressive disorder (MDD). Ketamine and electroconvulsive therapy (ECT) both exert rapid and potent antidepressive effects, and although these treatments may not act directly on the serotonin system, they both cause dose-dependent increases in serotonin levels, and there is convergent evidence suggesting that the serotonin system may be important for their mechanisms of action. In this study, we re-analysed a multi-centre dataset of 222 [^11^C]AZ10419369 PET measurements from three centres, including MDD patients examined with PET both before and after treatment with ketamine (*n* = 19 completers), saline placebo (*n* = 10), or ECT (*n* = 13 completers). Using a hierarchical Bayesian approach (SiMBA) that takes advantage of the full dataset to improve parameter estimation and enable data harmonisation across centres, we demonstrate large increases in 5-HT_1B_R binding following both ketamine (6.4%, 95% CI: 3.1–9.6%) and ECT (9.3%, 95% CI: 4.3–14.2%). Ketamine-induced changes were statistically distinguishable from placebo, and an exploratory cross-centre comparison enabled by the data harmonisation within the combined modelling framework, suggests that ECT-induced changes are also distinguishable from placebo. These changes were not associated with individual symptom improvement. These findings suggest that despite differences in the pri-mary target of these rapid acting treatments for depression, they may converge on similar downstream changes to the serotonin system.

## Introduction

The serotonin 1B receptor (5-HT_1B_R), like the serotonin 1A receptor (5-HT_1A_R), serves the role of both an inhibitory auto- and heteroreceptor in the brain. While 5-HT_1A_ autoreceptors are predominantly expressed within the raphe nuclei on serotonin cell bodies and dendrites, 5-HT_1B_ autoreceptors are located presynaptically on axon terminals in serotonin projection regions [1]. Activation of both of these receptor types is therefore able to regulate levels of extracellular serotonin. Although the role of 5-HT_1A_R in the pathophysiology of major depressive disorder (MDD) and its treatment has been studied in vivo using PET for many years across many studies [2–5], the role of the 5-HT_1B_R is less well characterised, partly due to an initial shortage of selective radioligands. This shortage may in turn have been related to the early belief that the 5-HT_1B_ receptor was not expressed in humans [e.g. 6]. With the development of [^11^C]P943 [7] and [^11^C]AZ10419369 [8], it is now possible to quantify 5-HT_1B_R in vivo using PET. The studies performed to date indicate that 5-HT_1B_R binding appears to be reduced in MDD [9–12], although these studies were conducted with relatively small sample sizes.

Studies examining the effects of treatment on 5-HT_1B_R binding in MDD patients have identified reductions in the dorsal brain stem following cognitive behavioural therapy [13] and escitalopram (a selective serotonin reuptake inhibitor, SSRI) [14] treatment. In contrast, studies of rapid-onset antidepressive treatments have identified increases in hippocampal 5-HT_1B_R binding following both ketamine [15] and electroconvulsive therapy, ECT [16], however the conclusions of these studies were limited by modest sample sizes. Specifically, [15] reported increases in hippocampal 5-HT_1B_R binding following ketamine, but these changes could not be differentiated from placebo owing to the small placebo group (*n* = 10). Similarly, [16] reported 5-HT_1B_R binding increases following ECT, but no placebo comparison was possible. Moreover, although both studies observed numerical increases in all regions, the changes were only statistically significant for the hippocampus where the signal to background ratio of [^11^C]AZ10419369 is low. If these treatment effects are genuine, we would expect that applying more powerful analytical methods to the same data would yield statistically distinguishable effects from placebo. These findings are, however, supported by preclinical studies showing that both ketamine [17, 18] and electrical stimulation [19] produce dose-dependent increases in extracellular serotonin concentrations.

We recently introduced a new approach for PET quantification and analysis called SiMBA (Simultaneous Multifactor Bayesian Analysis of PET Data), a hierarchical Bayesian approach for PET quantification and analysis that performs quantification of all recorded data simultaneously, exploiting similarities in parameter values both within and between individuals and regions [20]. We have recently extended this approach for application to non-invasive kinetic models [21]. In simulated [^11^C]AZ10419369 data, SiMBA reduced estimation error by up to 90% in some regions, and effectively halved the required sample size for detecting clinical treatment effects. Importantly, in addition to borrowing strength across the data and thereby exhibiting improved performance through incorporating additional data into the model, we could also show that SiMBA enables effective harmonisation of data collected at different PET centres [21].

Here, we sought to apply SiMBA to re-examine the effects of ketamine and ECT on 5-HT_1B_R binding, as well as to test whether these changes could be statistically distinguished both from one another as well as from the effects of the placebo arm of the ketamine study. Secondary aims included examining the regional specificity of treatment effects, evaluating differences in 5-HT_1B_R binding between MDD patients before treatment and healthy controls, as well as evaluating to what extent the inclusion of additional data from other studies and centres improves the precision of treatment effect estimates.

## Materials and methods

### Participants and measurement protocols

We analysed a combined sample of 222 [^11^C]AZ10419369 PET measurements performed at three different PET centres: Karolinska Institutet (KI) in Stockholm, Sweden; Nippon Medical School (NMS) in Tokyo, Japan; and the Neurobiology Research Unit at Rigshospitalet (NRU) in Copenhagen, Denmark (Table 1). These datasets have been described in detail previously [15, 16, 22, 23], and their combination and harmonisation using the non-invasive SiMBA model was described in [21].

**Table 1:** Summary statistics for different datasets.

Attribute	KI	NMS	NRU
**Acquisition**			
PET System	Siemens HRRT	Shimadzu Eminence SET-3000GCT/X	Siemens HRRT
**Sample**			
Total PET measurements	139	44	39
Unique individuals	99	31	39
Healthy volunteers, *n*	62	15	39
Age, years	39.9 ± 17.9	60.1 ± 14.8	30.1 ± 9.6
Sex, F/M	26/36	10/5	11/28
MDD patients, *n*	40	16	—
Age, mean *± *sd, years	40.8 ± 13.2	62.9 ± 14.6	
Sex, F/M	20/20	11/5	
**Treatment completers**^*a*^			
Ketamine, *n*	19^*b*^	—	—
Placebo, *n*	10	—	—
ECT, *n*	—	13	—
**Clinical characteristics**			
* Ketamine/Placebo arm*			
Medication-free, *n*	7	—	—
Medication-withdrawn, *n*	22	—	—
*ECT arm*			
ECT sessions, range	—	7–16	—

*KI* Karolinska Institutet (Sweden), *NMS* Nippon Medical School (Japan), *NRU* Neurobiology Research Unit (Denmark), *HRRT* high resolution research tomograph, *MDD* major depressive disorder, *ECT* electroconvulsive therapy. Age shown as mean *± *SD; F: female, M: male.

^*a*^Treatment completers had PET measurements both before and after treatment; these individuals are a subset of the MDD patients listed above. At KI, 10 MDD patients were measured without undergoing treatment intervention.

^*b*^One participant was excluded due to missing data during PET acquisition (see text).

Here, we provide a brief overview of their overall composition, highlighting the clinical variables which are the focus of this paper.

The KI data consists of measurements of healthy volunteers (HV) and MDD patients [11, 15, 22, 24], including repeated measurements of healthy volunteers from a test-retest study [22] as well as MDD patients before and after a single intravenous infusion with either racemic ketamine (0.5 mg/kg) or placebo [15]. The NMS data consists of measurements of HVs and MDD patients [16], with repeated measurements of MDD patients before and after ECT. The NRU consists of HV data obtained through the CIMBI database [23]. The datasets and their properties are described in Table 1. All patients met diagnostic criteria for a major depressive episode according to DSM-IV or DSM-5 criteria. Key exclusion criteria common to both treatment studies included psychotic disorder, substance abuse, organic brain disorder, and pregnancy; full inclusion and exclusion criteria, and study protocols for each treatment dataset are reported in the original publications [15, 16].

#### Within-subject pre- and post-treatment subsamples

The KI patients treated with ketamine (*n* = 20) and placebo (*n* = 10) were outpatients with SSRI treatment-resistant depression, defined as failure to respond to at least 4 weeks of SSRI treatment in adequate doses during the current depressive episode. All patients were psychotropic medication-free at the time of PET; patients who were on antidepressant treatment at the time of inclusion underwent supervised washout for a period corresponding to at least five half-lives of each drug prior to the baseline PET examination [15]. They underwent PET examinations both 1–3 days before, and 24–72 h after a single infusion of 0.5 mg/kg racemic ketamine or saline. Treatment allocation was randomised and double-blind [15]. The post-treatment measurement from one participant had to be removed from the analysis due to the subject having to leave the PET system for part of the acquisition, which caused missing time points, and prevented reliable estimation of the model for fitting the reference region time activity curve [21]. Thus, 19 patients in the ketamine arm and all 10 patients in the placebo arm had complete pre- and post-treatment data for analysis.

The NMS patients treated with ECT were hospitalised patients with major depressive episodes (*n* = 16 enrolled, *n* = 13 completers with both pre- and post-treatment PET scans), all of whom were on concurrent medication. Importantly, patients’ medication was in steady state at the time of PET, there were no changes in medication between their first and second PET measurements, and none of their medications act on 5-HT_1B_R. Concurrent antidepressant medications in the ECT group included mir-tazapine (*n* = 5), paroxetine (*n* = 4), duloxetine (*n* = 3), nortriptyline (*n* = 2), sertraline (*n* = 2), escitalopram (*n* = 1), and trazodone (*n* = 1), with several patients on combination regimens. Additional psychotropic medications included atypical antipsychotics (primarily quetiapine and olanzapine) and benzodiazepines. Full medication details and doses are reported in [16]. None of the patients received ketamine as part of their treatment or as an anaesthetic agent during ECT. These patients were hospitalised with severe major depressive episodes warranting ECT, and can be considered as difficult-to-treat, but no formal criterion was applied for treatment resistance. These patients were measured with PET before and up to one week after the ECT treatment series, which consisted of between 7 and 16 ECT treatments, 2–3 per week. The ECT study was open-label with no sham-ECT control [16].

### PET modelling and statistical analysis

We applied SiMBA using the simplified reference tissue model (SRTM) [25]. The model and the priors are more fully described in [21], with the priors more fully described in Supplementary Materials S1.

Clinical covariates were defined based on the constitution of the relevant dataset. In datasets that included data from patients, we included a covariate for patients with major depressive disorder (MDD) relative to healthy volunteers (HV), as well as an interaction between age and patient status, given the evidence for age-related declines in 5-HT_1B_R binding [21, 24, 26]. When pre-post intervention measurements were performed, we included covariates for whether the participant had been administered ECT, ketamine or placebo (relative to baseline) prior to PET; as well as their change in symptom score based on the 17-item Hamilton Depression Rating Scale (HAM-D) [27] or a short version of the Montgomery-Åsberg Depression Rating Scale, excluding items for appetite and sleep (MADRS-Short) [28]. The MADRS-Short scale was applied in the ketamine study because appetite is directly modulated by ketamine, and the short time interval between dosing and the second PET examination when they completed the scale did not allow for measuring changes in sleep. Hence, MADRS-Short scores were divided by 0.8 to estimate full MADRS scores [15], and then divided by 1.3 to convert to HAM-D-equivalent scores following the recommendations of [29] (i.e., MADRS-Short scores were divided by 1.04). All HAM-D and HAM-D-equivalent scores were divided by 10 to scale the unit response to represent a “clinically substantial” improvement for interpretability [30].

To account for centre differences, we included covariates for centre and centre × region interactions for the natural logarithms of *R*_1_, and *BP*_ND_; and for centre only for the natural logarithms of *k*′_2_, since this is a property of the reference region, and for the measurement error, *σ*, as described in [21]. We have previously shown that estimated associations between age and 5-HT_1B_R binding are highly consistent across the three PET centres when analysed both independently and in a combined harmonised model with covariates accounting for these differences, with highly correlated regional age effects between centres [21], suggesting that centre differences in baseline binding are effectively accounted for by these harmonisation covariates.

Statistical contrasts are presented using 80 and 95% credible intervals (CI) (or confidence intervals for frequentist analyses using linear mixed effects models for comparison) as well as the posterior probability of direction (*pd*) [31]. The *pd* represents the posterior probability that the parameter of interest is in the direction of its median, and so it varies between 0.5 and 1, where values close to 1 indicate a high probability of the existence of an effect, while values close to 0.5 indicate estimates whose poste-rior median is close to 0 and whose direction cannot be determined with any degree of certainty. For reference, a *pd* of 0.975 is most analogous to a two-sided frequentist *p*-value of 0.05, while a *pd* of 0.95 corresponds to a one-sided *p*-value of 0.05. Because we make use of Bayesian statistical analysis, these values can be interpreted as a continuous grade of evidence or probability, and so binary decision thresholds of 0.95 or 0.975, for example, can be considered to guide inferences using *pd*, but need not be considered as fixed boundaries. Given the inherent regularisation provided by hierarchical shrinkage, we did not apply additional corrections for multiple comparisons across regions [32] but interpret regional findings cautiously.

Sample sizes for this re-analysis were determined by the available data from the original studies; no prospective power calculation was performed for the present analysis. However, we have previously demonstrated in simulated data that SiMBA substantially improves statistical power relative to conventional methods, effectively halving required sample sizes for detecting treatment effects of this magnitude [21].

## Results

### Data harmonisation

Centre differences in the estimated parameters were estimated and corrected for within the SiMBA model relative to the KI model parameters. By correcting differences across all model parameters, centre differences are effectively accounted for at the level of the entire predicted time activity curve, as opposed to only the main parameter of interest within the model, which we have previously shown gives rise to consistent estimates of associations between age and 5-HT_1B_R binding in this dataset [21]. For differences in 5-HT_1B_R binding specifically, measured with the *BP*_ND_ parameter, centre differences were more modest between the KI and NRU data as expected because both datasets were acquired with Siemens HRRT PET systems (global *BP*_ND_ difference: +13.1%, 95% CI: 4.0–23.8%). For the NMS data, which was acquired with the lower resolution Shimadzu Eminence PET system, binding estimates were correspondingly lower (global *BP*_ND_ difference: −37.8%, 95% CI: −43.2–32.7%) because of higher degrees of spillout on average. Similarly, there was greater estimated region-specificity of differences for the NMS data compared to between the KI and NRU data (SD of centre × region corrections in log *BP*_ND_: 0.30 for NMS vs. 0.13 for NRU).

### Global clinical outcomes

**Fig. 1:**
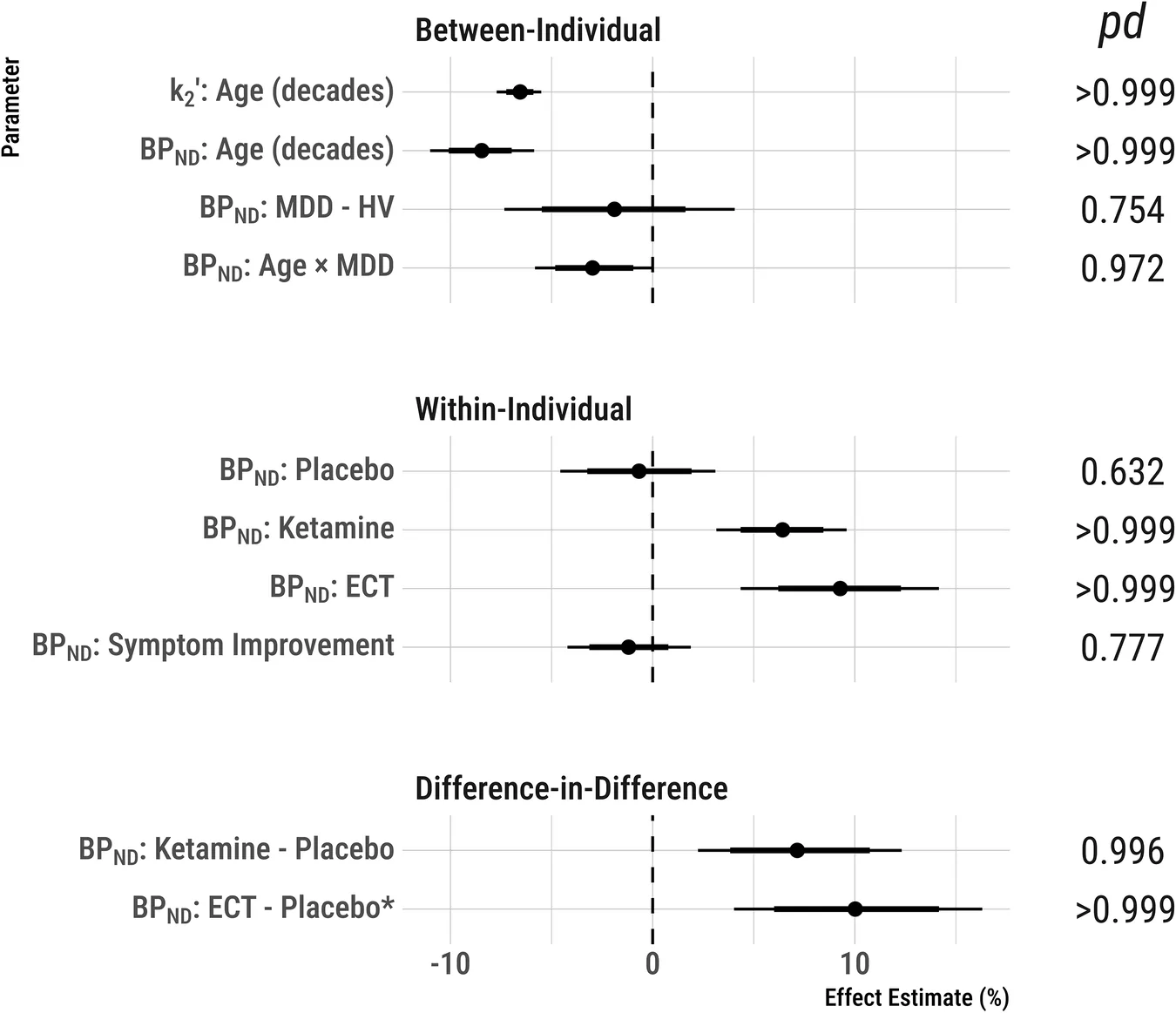
Clinical covariate estimates. The thick and thin intervals representing 80 and 95% CI respectively, and *pd* represents the posterior probability of direction which ranges from 0.5 (no evidence for a directional effect) to 1.0 (complete certainty of direction). For reference, *pd* = 0.95 is approximately analogous to a one-sided *p*-value of 0.05. MDD Major Depressive Disorder, HV Healthy volunteers, ECT Electroconvulsive therapy. * The ECT - Placebo comparison is made by comparing ECT to the placebo arm of the ketamine study, since sham-ECT was not administered in [16], as described in the text.

Estimated clinical covariates are shown in Fig. 1. As previously reported [21], there are between-individual decreases of both *k*_2_^′^ and *BP*_ND_ with increasing age. Although we did not include sex as a covariate in the main SiMBA model fit, we fit an additional model including sex, for which we showed no clear differences in binding (Female - Male: −2.4, 95% CI: −7.9–3.7%, *pd* = 0.805). We do not observe any evidence for a global effect of lower binding in MDD compared to controls (MDD - Control: −1.9, 95% CI: −7.3–4.1%, *pd* = 0.754), i.e. there is only a 75.4% posterior probability that the effect is less than zero. However, we observe evidence for an effect of age × MDD interaction (−3.0% greater reduction per decade 95% CI: −5.8–0.0%, *pd* = 0.972), suggesting that older MDD patients tend to exhibit lower binding than older controls. Based on the fact that the NMS patient group was on concurrent medication while the KI group was examined after drug washout, we also tested for effects of medication status between the two groups of patients, which did not show clear differences (Medicated - unmedicated: −1.2%, 95% CI: −10.1–7.8%, *pd* = 0.593).

Focusing on within-individual changes, clear global increases (i.e. across all examined brain regions) in 5-HT_1B_R binding are observed following both ketamine (6.4, 95% CI: 3.1–9.6%, *pd* > 0.999) and ECT (9.3, 95% CI: 4.3–14.2%, *pd* > 0.999) treatment, as opposed to placebo which yielded small to negligible changes (−0.7, 95% CI: −4.6–3.1%, *pd* = 0.632). 5-HT_1B_R changes induced by ECT were slightly larger than by ketamine treatment (*pd* = 0.973). Changes in *BP*_ND_ were not associated with any improvement in individual symptom scores over and above the average change in symptom scores.

Lastly, the difference-in-difference covariates show that the changes caused by both ketamine and ECT treatments are statistically distinguishable from those of placebo (ketamine: 7.1, 95% CI: 2.2–12.3%, *pd* = 0.996. ECT: 10.0, 95% CI: 4.0–16.3%, *pd* > 0.999). However, it should be noted that there was only one experimental placebo group, which was part of the ketamine trial: comparisons to placebo in the analysis of ECT effects are made with reference to the placebo arm of the ketamine study, which is why “ECT-placebo” is marked with an asterisk in Fig. 1. This comparison therefore makes the assumption that placebo-induced changes in *BP*_ND_ would be of a similar magnitude for sham-ECT as those observed for sham-ketamine if the former were to have been performed. Given the lack of change observed in the sham-ketamine group, we believe that this assumption is not unreasonable, but we leave this judgement to the reader.

### Regional heterogeneity

Although there was no overall global group difference in *BP*_ND_ between MDD patients and controls as described above (aside from age × MDD interaction effect), we also assessed whether there was any evidence for regional, as opposed to global, differences between MDD patients and controls. Although there was minimal variation between regions in their estimated deviations from the global effect in the MDD-HV contrast (SD in ∆ log BP_ND_ = 0.04), after combining the global and regional covariates, the 95% CI for the amygdala did not overlap with zero. This suggests that amygdalar 5-HT_1B_R binding may be lower in patients with MDD compared to controls (estimated ∆BP_ND_ = −6.3%, 95% CI: −11.7–0.3%, *pd* = 0.985). We recommend that this finding be interpreted cautiously given the modest effect size and exploratory nature of the regional analysis. Results for all regions are shown in Supplementary Materials S2.

When comparing the effects of treatments, no region was clearly statistically differentiable from the mean treatment effect for ECT or ketamine. For ECT, the hippocampus had the largest proportional changes (+6.2% relative to the mean effect of ECT treatment, *pd* = 0.937), and the frontal cortex had the smallest changes (−3.9% relative to the mean ECT treatment effect, *pd* = 0.907). For ketamine, all differences from the mean were closer to 0 than for ECT (all *pd* < 0.65). The standard deviation of the random effect deviations from the mean treatment effects were over three times greater for ECT (SD ∆ log *BP*_ND_ = 0.044) than for ketamine (SD ∆ log *BP*_ND_ = 0.013), corresponding to a *pd* = 0.90 that ECT causes more regionally heterogeneous effects on 5-HT_1B_R binding than ketamine does. Results for all regions are shown in Supplementary Materials S2.

### Comparison with conventional methods and benefits of data pooling

**Fig. 2:**
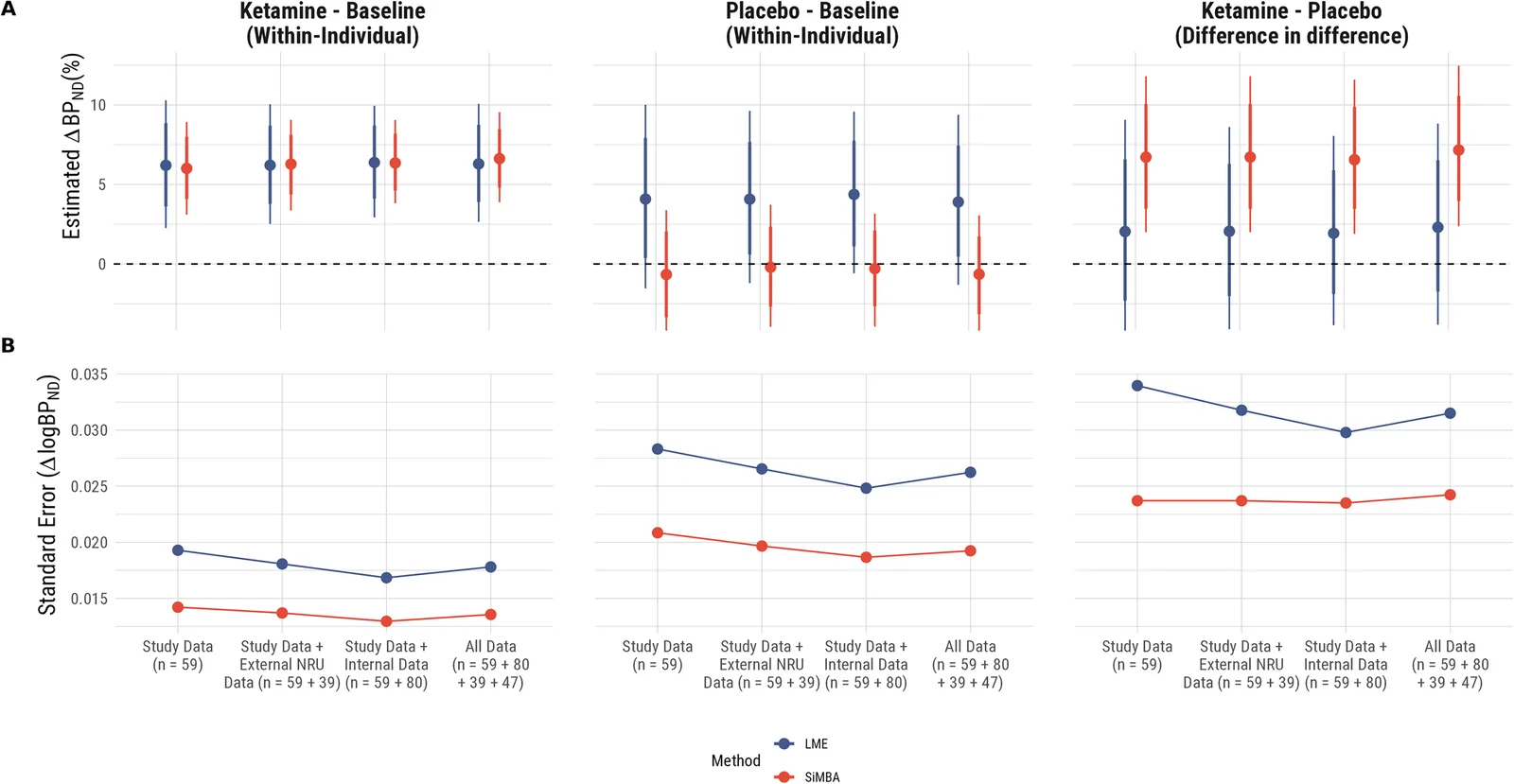
Estimated changes in *BP*_ND_: evaluation of improvements to inferences using different analysis methods and the effects of including additional data from participants who did not undergo treatment. Blue represents conventional quantification followed by linear mixed effects (LME) analysis, while red represents SiMBA. For each comparison, we make the same inferences using the study data, but by including additional data collected at another centre (External) or the same centre (Internal) within the model alongside the data from the original study. In **A**, the difference estimates are shown with thick and thin intervals representing 80 and 95% uncertainty intervals respectively, credible intervals in red and confidence intervals in blue. In **B**, the SE of the estimates is displayed. In all cases, *n* represents the number of PET measurements included in the analysis.

We were previously able to show in simulated data that SiMBA exhibited better accuracy and statistical power than conventional analysis, and that this method could effectively harmonise data from different research centres, with highly consistent age-related effects on 5-HT_1B_R binding [21]. Owing to the ability of hierarchical models to exploit similarities in the underlying parameter distributions to improve performance, we were also able to show that estimation accuracy improved with increasing sample sizes using SiMBA for both invasive [20] and noninvasive [21] models. Therefore, we aimed to compare the means and standard errors (SE) of estimated clinical contrasts to evaluate the impact of these methodological factors. First, we compared the results using SiMBA with those derived using conventional methods. Secondly, we tested whether the inclusion of additional data from subjects not belonging to the contrast of interest, which we will refer to as *ancillary participant data*, within the greater analysis model is able to improve the SE of the estimated clinical contrasts further. To this end, we sought to examine the estimated contrasts of [15] by measuring the effects of ketamine and placebo, comparing the means and SE of their estimates. We estimated these contrasts with both SiMBA and conventional methods, using the original dataset as well as in expanded datasets with ancillary participant data from other studies or centres. The results of these analyses are shown in Fig. 2.

Firstly, comparing the performance of SiMBA (red) and conventional methods (blue), the means of the estimated contrasts are shown in Fig. 2A. For the ketamine–baseline contrast (left column), the means were broadly similar across all datasets, however we observed striking differences in the means of the placebo–baseline contrast (middle column), which were also reflected in the ketamine–placebo contrast (right column). Importantly, although changes in 5-HT_1B_R binding after placebo were not significantly different from zero using conventional methods, they were nevertheless reasonably large (approximately two thirds the size of the effect of ketamine). As a result, this caused the ketamine–placebo contrast to be estimated as smaller using conventional methods. However, using SiMBA, the estimated effect of placebo on 5-HT_1B_R binding is almost exactly centred at zero, resulting in greater sensitivity for detecting ketamine–placebo difference-in-difference effects. By comparing the credible intervals (Fig. 2A) and SE of the estimates (Fig. 2B), it is clear that SiMBA estimates have better precision (i.e. lower SE) for all comparisons across all contrasts and all datasets relative to conventional methods. These differences in the SE are greater for the placebo–baseline contrast compared to the ketamine–baseline contrast, which is expected owing to the smaller number of participants who received placebo than who received ketamine. This improved precision is also reflected in the ketamine–placebo contrast, which also gives rise to improved sensitivity for ketamine–placebo effects. Notably, for the ketamine–placebo differences estimated using conventional methods, even if they were to have the same mean estimate as those of the SiMBA analysis, their wide confidence intervals would still overlap with zero, resulting in non-significant outcomes. Hence, the improved sensitivity of SiMBA for statistically distinguishing treatment and placebo effects is attributable both to improvements to the mean and standard error of the estimates.

In Fig. 2B, it is clear that the inclusion of ancillary participant data can improve the precision of estimated differences in a given study, using either SiMBA or linear mixed-effects models. For both the ketamine–baseline and placebo–baseline contrasts, the SE was improved through incorporating ancillary participant data collected at the same PET centre using the same PET system, and even by ancillary data collected using the same PET system at a different PET centre with different frame sequences and preprocessing. The results for the ECT–baseline contrasts are provided in Supplementary Materials S3. For the combined dataset, the SE was slightly higher than for the internal data, which is presumably caused by the model having to account for the different PET system used in the NMS dataset, or alternatively differences in the covariates relating to symptom improvement with changes to *BP*_ND_ after treatment due to the inclusion of an additional treatment group. Despite the small increase in the SE of the estimate, the fact that the model is applied to more subjects from a wider background can be seen to afford the model greater generality. Furthermore, although these changes are small, especially in comparison to the difference between using SiMBA and using conventional methods, we speculate that the addition of additional controls would likely have a greater impact if the original study data were smaller.

### Prior sensitivity

We assessed the sensitivity of estimated parameters to the specification of prior distributions using power-scaling sensitivity analysis [33], which uses importance sampling to approximate the posterior under strengthened and weakened priors and likelihoods without having to re-fit the model. All 6887 estimated parameters exhibited high likelihood sensitivity (*>* 0.05), and likelihood sensitivity was substantially greater than prior sensitivity in all cases, meaning that the posterior estimates were predominantly determined by the data rather than the priors. Several parameters exhibited prior sensitivity (*>*0.05), indicating some degree of tension between the prior and the data, though the likelihood remained dominant. The strongest of these was the centre deviation in *BP*_ND_ for the NMS data compared to KI, for which we intentionally defined a regularising prior penalising larger differences. In other words, with a less informative prior, the model would have estimated greater differences in mean *BP*_ND_ between the NMS and KI datasets, which were restricted by our prior. However, the practical significance of this sensitivity was limited: under power-scaling of the prior from *α* = 0.8 (weakening) to *α* = 1.25 (strengthening), corresponding to increasing or decreasing the standard deviation of a normally-distributed prior by approximately 11–12%, the estimated mean difference of −37.5% varied only between −39.3 and −36.2%, respectively. For all clinical covariates of *BP*_ND_, strengthening or weakening the prior by the same degree changed the posterior estimates by less than 0.5% of the mean. For the placebo effect in particular, the estimated effect of −0.7% varied only between −0.8 and −0.4%.

## Discussion

In this study, we re-analysed the data collected at three different PET centres comprising the largest single analysis of [^11^C]AZ10419369 PET data to date. This data was modelled using SiMBA, which not only improves the quantitative accuracy and power in the original datasets, but also enables efficient harmonisation of estimated parameters between the data collected at the different centres to improve its performance further and allow new comparisons which could not previously be made. We show that ECT and ketamine cause large increases in 5-HT_1B_R binding in all examined regions of the brain, which can be statistically distinguished from the effects of placebo. Although both treatments result in global increases in 5-HT_1B_R binding, our model indicates that ECT produces changes with greater regional heterogeneity than ketamine does. Despite the large reductions in the MDD rating scales after ketamine or ECT treatment [15, 16], the degree to which symptoms improved was not associated with any additional explanatory potential for change in 5-HT_1B_R binding. Addressing previously reported differences between MDD patients and controls in 5-HT_1B_R, we did not find evidence of a general difference between MDD patients and controls, although we did observe lower [^11^C]AZ10419369 binding in the amygdala of MDD patients compared to controls. However, our model indicated a reasonably high probability of there being lower 5-HT_1B_R binding in the amygdala in patients compared to controls, as well as for an interaction between diagnosis and age, with lower binding in the older MDD patient groups.

One of the most important differences between the present analysis and previous analyses of these data is in the estimation of the effect of the placebo arm of the ketamine study. Accounting for the effects of placebo are of particular importance for studying MDD [34], however it is also extremely costly to collect large placebo groups when using such an expensive measure as PET. Using conventional analysis [15], showed relatively large *numerical* increases in binding following ketamine which were significant in the hippocampus, but not statistically significantly different from placebo. Due to the small number of participants who underwent placebo treatment (*n* = 10), these estimates had a high level of uncertainty and therefore the confidence intervals for a greater increase in 5-HT_1B_R binding after ketamine compared to placebo overlapped with zero. Although placebo treatment caused reductions in symptom scores, and even resulted in one participant experiencing full remission [15], placebo intervention is not thought to produce any systematic changes in 5-HT_1B_R binding. However, using SiMBA, the placebo effects estimates were much more precise (Fig. 2), and estimated to be almost exactly centred around zero, as expected a priori. As a result, although there was previously insufficient evidence to identify these changes, using SiMBA it is now possible to statistically differentiate the effects of ketamine from those of placebo with a high degree of certainty. Furthermore, although the placebo intervention was only part of the ketamine treatment study, by analysing the data together in a combined model that accounts for the differences between centres, we were able to examine to what extent the effects of ketamine and ECT treatment can be distinguished from the placebo intervention of the ketamine study. Because ECT was administered at NMS while ketamine and placebo were administered at KI, it should be noted that centre and treatment effects are partially confounded. However, treatment effects are estimated as within-individual pre-post changes, and centre differences were accounted for through covariates for all model parameters underlying the estimated time activity curve, as opposed to only the focal binding parameter, which should largely mitigate these differences. If we further assume that the expectancy effects of both placebo interventions cause similar changes to brain 5-HT_1B_R binding, which we consider highly likely, and considering that placebo effects on binding were centred around zero, then we can conclude that the effects of ECT on 5-HT_1B_R binding are likely also statistically distinguishable from those of sham-ECT. However, it should also be acknowledged that expectancy effects, physiological stress responses, and treatment context differ markedly between intravenous saline infusion and ECT procedures, and these factors could theoretically influence serotonergic systems differently.

Considering that this is a re-analysis of previously collected data which uncovers novel outcomes which were not evident in the original analysis, it is worth considering the basis for these different conclusions. SiMBA is a Bayesian nonlinear hierarchical, or mixed-effects, model, which can “borrow strength” from data across all individuals in the sample at once. Such models are common in classic pharmacokinetics [35], but still quite novel in PET pharmacokinetic modelling [20, 36–38]. We have shown in simulated data that SiMBA is able to dramatically improve both the accuracy of estimated parameters using SRTM together with the statistical power for the inferences drawn using this data, thereby reducing required sample sizes for answering the same research questions. We also showed in real data that the inferences drawn using SiMBA examining the relationship between age and 5-HT_1B_R binding in each of the separate datasets were replicable and highly consistent in magnitude, despite differences in age ranges and PET acquisition between research centres. For these reasons, we believe that the present results are likely to be more precise than those of the original studies, and potentially more generalisable on the basis of being applied to data from multiple centres and to a wider range of individuals. However, it should also be noted that the biological conclusions depend on the modelling assumptions, and independent replication with new data would further strengthen confidence in these findings.

ECT and ketamine are both effective treatments that are used for treatment-resistant MDD [39, 40]. Although a recent trial demonstrated non-inferiority of ketamine compared to ECT [41], ECT has been found to be more effective in hospitalised MDD patients [42] and the former study has been subject to methodological criticisms [43]. While most pharmacological treatments for MDD act through inhibition of the serotonin transporter [44], ECT and ketamine have more generalised effects without targeting the serotonin transporter [45]. However, there is evidence suggesting that these treatments may also cause increases in brain serotonin levels. Electrical stimulation in anaesthetised rodents produces pronounced elevations in extracellular serotonin levels [19, 46]. In patients treated with serotonin reuptake inhibitors, two case studies have reported cases of serotonin syndrome occurring after ECT, although this is extremely rare in clinical practice [47, 48]. Ketamine has also been shown to produce dose-dependent increases in brain serotonin concentrations in rodents [17, 49, 50]. Moreover, one study even found that prior endogenous serotonin depletion could abolish the antidepressant effects of S-ketamine in an animal model of depression, which could be rescued with a 5-HT_1B_R agonist [51]. The current results strengthen the association between both ECT and ketamine treatment and the serotonin system in humans, demonstrating that both of these treatments produce large global elevations in 5-HT_1B_R binding of similar magnitude, despite their different mechanisms of action. Given the lack of association between changes in symptom scores and global elevations in 5-HT_1B_R binding, we speculate that it is likely that 5-HT_1B_R changes are a downstream effect of the treatment-induced changes in serotonin levels. The lack of association between 5-HT_1B_R binding changes and individual symptom improvement warrants consideration. This dissociation suggests that the observed receptor changes may represent a downstream or epiphenomenal consequence of treatment rather than a direct mediator of therapeutic efficacy. Alternatively, the binding changes may reflect a necessary but not sufficient condition for clinical improvement, or may relate to aspects of treatment response not captured by standard symptom scales. Similar dissociations between neurobiological changes and clinical outcomes have been reported for hippocampal volume increases following ECT [52].

Although we observed increased 5-HT_1B_R binding following treatment, more work is necessary to clarify exactly what these increases represent. PET cannot distinguish between binding to 5-HT_1B_R autoreceptors and heteroreceptors, and indeed changes in binding to either one could reflect altered receptor density, changes in receptor affinity states, and/or reduced competition from endogenous serotonin. Given that acute serotonin release would be expected to decrease rather than increase *BP*_ND_, and that our measurements were performed days after treatment, receptor-level changes seem more plausible than sustained alterations in extracellular serotonin. However, the observed increases could be be explained by either upregulation of receptors or an increased proportion of receptors in the active conformation for which [^11^C]AZ10419369 would have greater affinity since it is a partial agonist for the 5-HT_1B_R [53]. In either case, we speculate that the observed increases in binding may reflect an upregulation of inhibitory 5-HT_1B_R autoreceptor density, or action, in order to reduce serotonin release and thereby maintain brain homeostasis.

Although the changes caused by ECT and ketamine were global, we observed a greater regional heterogeneity of the changes caused by ECT compared to those of ketamine treatment. In particular, ECT-induced changes were highest in the hippocampus (Supplementary Materials S2). This is explained by the fact that the effects of ECT treatment are based on an induced seizure that is believed to emanate from subcortical foci, particularly the hippocampi, which spreads to the rest of the brain [54]. Increases in hippocampal volume after ECT have been replicated in numerous independent cohorts [55, 56], and notably also not related to clinical improvement, but were associated with cognitive side-effects [52]. In contrast, ketamine is assumed to be distributed equally throughout the brain through the blood system, resulting in more spatially homogeneous changes.

The consistency of increases in 5-HT_1B_R binding following both ketamine and ECT as rapid-acting antidepressant treatments is most striking when considered in contrast to the effects of delayed-onset antidepressant treatments. In MDD patients, chronic treatment with escitalopram [14] and cognitive behavioural therapy (CBT) [13] both resulted in decreases in 5-HT_1B_R binding in the dorsal brain stem (DBS, which consists primarily of the dorsal and median raphe nuclei). In the former study, these changes were also associated with alleviation of depressive symptoms [14]. Even a single acute clinically relevant dose of SSRI medication was shown to produce decreases in 5-HT_1B_R binding in this region, in both healthy controls [57] and in non-human primates [58], together with increases in projection regions. This contrasts starkly with the clear *increases* in 5-HT_1B_R binding following ECT and ketamine treatment, which are found not only in the DBS but in all regions examined. Taken together, alterations to 5-HT_1B_R binding appear to be a common factor underlying multiple different MDD treatments, although there appears to be a dissociation in their effects specifically in the raphe nuclei that differs between – but is common within – rapid-onset and delayed-onset MDD treatments.

The clinical relevance of treatment-induced increases in 5-HT_1B_R binding remains to be established. Given the predominantly presynaptic localisation of 5-HT_1B_R, where they function as inhibitory autoreceptors regulating serotonin release, the dissociation in these changes are difficult to reconcile within a simple mechanistic framework. One possibility is that the increases observed after ketamine and ECT represent a home-ostatic response to treatment-induced serotonin release, while decreases after SSRIs reflect adaptation to sustained serotonin transporter blockade. Further mechanistic studies are needed to understand the functional consequences of these receptor changes and their relationship to therapeutic effects.

Global reductions in [^11^C]AZ10419369 binding have been observed following acute elevations of endogenous serotonin induced by fenfluramine administration [53, 59], indicating that [^11^C]AZ10419369 *BP*_ND_ is sensitive to fluctuations in endogenous serotonin levels. However, these effects have only been shown acutely after treatment, after minutes to hours. We interpret increases or decreases in [^11^C]AZ10419369 *BP*_ND_ observed over days to more likely reflect changes in 5-HT_1B_ receptor levels rather than sustained alterations in extracellular serotonin concentrations. This view is supported by preclinical studies showing that both ketamine [17, 18] and electrical stimulation [19] produce dose-dependent increases, not decreases, in extracellular serotonin concentrations.

In light of the apparent discrepancy between rapid-onset and delayed-onset antidepressant medications, it is noteworthy that both rapid-onset treatments are non-selective, causing widespread changes in the brain. Ketamine acts on the glutamate system, the main excitatory neurotransmitter in the mammalian brain, and ECT acts by causing a generalised seizure, which is also inherently linked to glutamate by virtue of the fact that glutamate neurons are by far the most common neurons in the brain. In contrast, delayed-onset pharmacological treatments tend to act primarily on the 5-HT system. As such, it would be of clear value to evaluate whether other more pharmacologically specific rapid-onset antidepressant medications cause changes in 5-HT_1B_R which more closely resemble those of ECT and ketamine or those of delayed-onset treatments.

Studies in rodents suggest that agonism of the 5-HT_1B_R directly also demonstrates efficacy as a potent rapid-onset antidepressant treatment [12, 60]. Therefore, it would be valuable to evaluate the potential of 5-HT_1B_R agonists for treatment of MDD in humans, where today non-selective 5-HT_1B_R agonists such as psilocin, the active metabolite of psilocybin, hold promise for rapid antidepressive effects, even in treatment resistant patients [61–63]. It also remains to be determined what is causing the observed changes to 5-HT_1B_R binding, and whether there could be one pathway in common in both ECT and ketamine, or perhaps even in all antidepressant treatments in general. Preclinical studies focusing on 5-HT_1B_R changes following diverse antidepressant treatments and how these can be blocked, as well as whether blocking the 5-HT_1B_R changes also blocks behavioural improvements, may be able to shed light on these mechanisms.

## Supplementary information

Supplemental Material

## Data Availability

The R code used to apply SiMBA is provided in an open repository (https://github.com/mathesong/SiMBA_Ref_Materials), including a simulated dataset and an anno-tated notebook demonstrating the method. The measured data used in this study are drawn from previously published studies [11, 15, 16, 22, 24].
