# Supplementary material for: Convergent increases in serotonin 1B receptor binding following ketamine and electroconvulsive therapy: a multi-centre bayesian re-analysis of PET data
Source: Mol Psychiatry. 2026 Jul 8;31(11):6351–9. doi: 10.1038/s41380-026-03704-2 (PMC13668655; doi:10.1038/s41380-026-03704-2)
Supplement: Supplementary file 1 [file 41380_2026_3704_MOESM1_ESM.pdf]

6 SUPPLEMENTARY MATERIALS

6.1 Supplementary Materials S1: Prior Definitions

Overview and parameterisation

All priors are specified on the natural logarithm of the SRTM parameters such that all additive deviations correspond with proportional differences:

$$\log R_1, \quad \log k_2', \quad \log BP_{ND}.$$

Region and centre effects use dummy coding with frontal cortex as the regional reference level and KI as the centre reference level. Age is centred and scaled per decade. Clinical symptom scores (HAM-D or MADRS converted as described in the main text) are divided by 10 so that a unit reflects a clinically substantial change. Treatment contrasts are defined relative to baseline (placebo, ketamine, ECT).

Global intercepts

$$\alpha_{\log R_1} \sim \text{Normal}(0, 0.25), \quad \alpha_{\log k_2'} \sim \text{Normal}(-2, 0.25), \quad \alpha_{\log BP_{ND}} \sim \text{Normal}(0, 0.25).$$

Intercepts are interpreted at the reference level for region and centre, with covariates at 0.

6.1.1 Individual deviations

Differences between individuals were defined by specifying the primary pharmacokinetic parameters in one variance-covariance matrix.

$$\begin{bmatrix} \tau_{\text{R}_1} \\ \tau_{\text{k}_2'} \\ \tau_{\text{BP}_{\text{ND}}} \end{bmatrix} \sim \text{MVNormal}\left(\begin{bmatrix} 0 \\ 0 \\ 0 \end{bmatrix}, \Sigma_{\text{Subject}} \right)$$

$$\Sigma_{\text{Subject}} = D_{\text{Subj}} \, \mathbf{R}_{\text{Subj}} \, D_{\text{Subj}}$$

$$D_{\text{Subj}} = \text{diag}\Big(\sigma_{\text{R}_1}, \; \sigma_{\text{k}_2'}, \; \sigma_{\text{BP}_{\text{ND}}}\Big)$$

Here D_{Subj} is a diagonal matrix of standard deviations, and $\mathbf{R}_{\text{Subj}}$ is a correlation matrix drawn from an LKJ prior. Together these define the covariance structure for subject-level deviations.

$$\begin{aligned} \sigma_{\text{R}_1} &\sim \text{Half-Normal}(0, \; 0.3) \\ \sigma_{\text{k}_2'} &\sim \text{Half-Normal}(0, \; 0.1) \\ \sigma_{\text{BP}_{\text{ND}}} &\sim \text{Half-Normal}(0, \; 0.3) \\ \mathbf{R}_{\text{Subj}} &\sim \text{LKJ}(1) \end{aligned}$$

Region effects

For $\log BP_{ND}$ and $\log R_1$, region effects are unpooled, zero-centred:

$$\upsilon_{j,\log R_1} \sim \text{Normal}(0, 0.3), \quad \upsilon_{j,\log BP_{ND}} \sim \text{Normal}(0, 0.3).$$

For $\log k_2'$, region effects are partially pooled:

$$\upsilon_{j,\log k_2'} \sim \text{Normal}(0, \sigma_{\log k_2',\text{reg}}), \quad \sigma_{\log k_2',\text{reg}} \sim \text{Half-Normal}(0, 0.1).$$

PET × Region (within-individual) deviations

Highly constrained deviations capture PET-to-PET and region-within-individual variability:

$$\begin{bmatrix} \phi_{\log R_1} \\ \phi_{\log k_2'} \\ \phi_{\log BP_{ND}} \end{bmatrix} \sim \text{MVNormal}\left(\begin{bmatrix} 0 \\ 0 \\ 0 \end{bmatrix}, \, \Sigma_{\text{TAC}} \right), \quad \Sigma_{\text{TAC}} = D_{\text{TAC}} \, \mathbf{R}_{\text{TAC}} \, D_{\text{TAC}},$$

with

$$\sigma_{\log R_1}, \sigma_{\log k_2'}, \sigma_{\log BP_{ND}} \sim \text{Half-Normal}(0, 0.025), \quad \mathbf{R}_{\text{TAC}} \sim \text{LKJ}(2).$$

Centre main effects and Region × Centre
Centre shifts on parameter means:

$$\beta_{\log R_1,\mathrm{Centre}},\ \beta_{\log k_2',\mathrm{Centre}},\ \beta_{\log BP_{ND},\mathrm{Centre}}\ \sim\ \mathrm{Normal}(0,0.1).$$

Region-within-centre interactions for $\log R_1$ and $\log BP_{ND}$:

$$\beta_{\log R_1,\mathrm{Centre}\times\mathrm{Region}},\ \beta_{\log BP_{ND},\mathrm{Centre}\times\mathrm{Region}}\ \sim\ \mathrm{Normal}(0,0.1).$$

Clinical and age covariates
Global slopes:

$$\beta_{\log BP_{ND},\mathrm{Age}}\sim\mathrm{Normal}(0,0.1),\quad \beta_{\log k_2',\mathrm{Age}}\sim\mathrm{Normal}(0,0.1),$$

$$\beta_{\log BP_{ND},\mathrm{MDD}},\ \beta_{\log BP_{ND},\mathrm{Placebo}},\ \beta_{\log BP_{ND},\mathrm{Ketamine}},\ \beta_{\log BP_{ND},\mathrm{ECT}},\ \beta_{\log BP_{ND},\Delta\mathrm{Symptoms}}\ \sim\ \mathrm{Normal}(0,0.2).$$

Region-varying slopes (random slopes) on $\log BP_{ND}$:

$$\beta_{j,\mathrm{Age}}\sim\mathrm{Normal}(\beta_{\log BP_{ND},\mathrm{Age}},\sigma_{\mathrm{Age}}),\quad \sigma_{\mathrm{Age}}\sim\mathrm{Half\text{-}Normal}(0,0.05),$$

$$\beta_{j,\mathrm{MDD}},\ \beta_{j,\mathrm{Ketamine}},\ \beta_{j,\mathrm{ECT}}\sim\mathrm{Normal}(0,0.05).$$

Observation model (heteroscedasticity)
The log residual SD uses a semi-parametric structure with centre-specific smooths over TAC time and covariates that affect precision (frame duration, region size, injected radioactivity), as described in Matheson et al. (2025).

Correlations
Correlation structures follow LKJ (Lewandowski et al., 2009) priors:

$$\mathrm{cor(ID\ block)}\sim\mathrm{LKJ}(1),\qquad \mathrm{cor(PET{:}Region\ block)}\sim\mathrm{LKJ}(2).$$

Notes on prior choice
Priors are weakly to moderately informative and centred at biologically plausible values. Scales were chosen to regularise ill-posed directions in the nonlinear SRTM while allowing region and centre adjustments when supported by data. Age and clinical priors are tighter than treatment-effect priors only where strong prior shrinkage aids identifiability.

Prior specification used
In R code using the `brms` package, the code for defining the model equation and priors is as follows:

```
formula <- bf(
  TAC ~ srtm_model(logR1, logk2prime, logBPnd,
                   t_tac, t0,
                   A, B, C,
                   alpha, beta, gamma,
                   Ph1, Th1),

  # Heteroscedastic observation model for log(sigma)
  lf(sigma ~ 1 + s(t_tac, by=Centre) +
                 Centre +
                 InjRad_logc +
                 dur_logc +
                 logRegSize_c +
                 (0 + logRegSize_c | Centre) +
                 (1 | Region:Centre) +
                 (1 | PET),
     center = FALSE),

  # Nonlinear parameters and their linear predictors
  logR1 ~ 1 + Centre*Region + (1|k|ID) + (1|l|PET:Region),
  logk2prime ~ 1 + Age_dec_c + Centre + (1|m|Region) +
               (1|k|ID) + (1|l|PET:Region),
  logBPnd ~ 1 + Centre*Region +
```

```
            Age_dec_c + (0 + Age_dec_c | Region) +
            DiagnosisMDD + (0 + DiagnosisMDD | Region) +
            TreatmentPlacebo +
            TreatmentKetamine + (0 + TreatmentKetamine | Region) +
            TreatmentECT + (0 + TreatmentECT | Region) +
            delta_Symptoms +
            (1|k|ID) + (1|l|PET:Region),

  # Nonlinear fit
  nl = TRUE, center = TRUE
)

# Full prior set with explanatory headers
mlsrtm_prior_clin <- c(

  # Global intercepts on log scale
  set_prior("normal(0, 0.25)",  nlpar = "logR1"),
  set_prior("normal(-2, 0.25)", nlpar = "logk2prime"),
  set_prior("normal(0, 0.25)",  nlpar = "logBPnd"),

  # Between-individual SDs and correlations (ID block)
  set_prior("normal(0, 0.3)", nlpar = "logR1",     class = "sd", group = "ID"),
  set_prior("normal(0, 0.1)", nlpar = "logk2prime",class = "sd", group = "ID"),
  set_prior("normal(0, 0.3)", nlpar = "logBPnd",   class = "sd", group = "ID"),
  set_prior("lkj(1)", class = "cor", group = "ID"),

  # PET:Region deviations (within-individual, highly constrained)
  set_prior("normal(0, 0.025)", nlpar = "logR1",     class = "sd", group = "PET:Region"),
  set_prior("normal(0, 0.025)", nlpar = "logk2prime",class = "sd", group = "PET:Region"),
  set_prior("normal(0, 0.025)", nlpar = "logBPnd",   class = "sd", group = "PET:Region"),
  set_prior("lkj(2)", class = "cor", group = "PET:Region"),

  # Region effects (unpooled) for logR1 and logBPnd; FC is reference
  set_prior("normal(0, 0.3)", coef="RegionACC",  nlpar="logR1"),
  set_prior("normal(0, 0.3)", coef="RegionAMG",  nlpar="logR1"),
  set_prior("normal(0, 0.3)", coef="RegionDBS",  nlpar="logR1"),
  set_prior("normal(0, 0.3)", coef="RegionHIP",  nlpar="logR1"),
  set_prior("normal(0, 0.3)", coef="RegionINS",  nlpar="logR1"),
  set_prior("normal(0, 0.3)", coef="RegionOC",   nlpar="logR1"),
  set_prior("normal(0, 0.3)", coef="RegionTHA",  nlpar="logR1"),
  set_prior("normal(0, 0.3)", coef="RegionVSTR", nlpar="logR1"),

  set_prior("normal(0, 0.3)", coef="RegionACC",  nlpar="logBPnd"),
  set_prior("normal(0, 0.3)", coef="RegionAMG",  nlpar="logBPnd"),
  set_prior("normal(0, 0.3)", coef="RegionDBS",  nlpar="logBPnd"),
  set_prior("normal(0, 0.3)", coef="RegionHIP",  nlpar="logBPnd"),
  set_prior("normal(0, 0.3)", coef="RegionINS",  nlpar="logBPnd"),
  set_prior("normal(0, 0.3)", coef="RegionOC",   nlpar="logBPnd"),
  set_prior("normal(0, 0.3)", coef="RegionTHA",  nlpar="logBPnd"),
  set_prior("normal(0, 0.3)", coef="RegionVSTR", nlpar="logBPnd"),

  # Region effects (pooled) for logk2prime
  set_prior("normal(0, 0.1)", nlpar="logk2prime", class="sd", group="Region"),

  # Centre main effects (KI is reference)
  set_prior("normal(0, 0.1 )", coef="CentreNMS", nlpar="logR1"),
  set_prior("normal(0, 0.1 )", coef="CentreNRU", nlpar="logR1"),
  set_prior("normal(0, 0.1 )", coef="CentreNMS", nlpar="logk2prime"),
  set_prior("normal(0, 0.1 )", coef="CentreNRU", nlpar="logk2prime"),
  set_prior("normal(0, 0.1 )", coef="CentreNMS", nlpar="logBPnd"),
  set_prior("normal(0, 0.1 )", coef="CentreNRU", nlpar="logBPnd"),

  # Region x Centre interactions for logR1 and logBPnd
  set_prior("normal(0, 0.1)", coef="CentreNRU:RegionACC", nlpar="logR1"),
  set_prior("normal(0, 0.1)", coef="CentreNRU:RegionAMG", nlpar="logR1"),
  set_prior("normal(0, 0.1)", coef="CentreNRU:RegionDBS", nlpar="logR1"),
  set_prior("normal(0, 0.1)", coef="CentreNRU:RegionHIP", nlpar="logR1"),
  set_prior("normal(0, 0.1)", coef="CentreNRU:RegionINS", nlpar="logR1"),
  set_prior("normal(0, 0.1)", coef="CentreNRU:RegionOC",  nlpar="logR1"),
  set_prior("normal(0, 0.1)", coef="CentreNRU:RegionTHA", nlpar="logR1"),
```

```
  set_prior("normal(0, 0.1)", coef="CentreNRU:RegionVSTR",nlpar="logR1"),

  set_prior("normal(0, 0.1)", coef="CentreNMS:RegionACC", nlpar="logR1"),
  set_prior("normal(0, 0.1)", coef="CentreNMS:RegionAMG", nlpar="logR1"),
  set_prior("normal(0, 0.1)", coef="CentreNMS:RegionDBS", nlpar="logR1"),
  set_prior("normal(0, 0.1)", coef="CentreNMS:RegionHIP", nlpar="logR1"),
  set_prior("normal(0, 0.1)", coef="CentreNMS:RegionINS", nlpar="logR1"),
  set_prior("normal(0, 0.1)", coef="CentreNMS:RegionOC",  nlpar="logR1"),
  set_prior("normal(0, 0.1)", coef="CentreNMS:RegionTHA", nlpar="logR1"),
  set_prior("normal(0, 0.1)", coef="CentreNMS:RegionVSTR",nlpar="logR1"),

  set_prior("normal(0, 0.1)", coef="CentreNRU:RegionACC", nlpar="logBPnd"),
  set_prior("normal(0, 0.1)", coef="CentreNRU:RegionAMG", nlpar="logBPnd"),
  set_prior("normal(0, 0.1)", coef="CentreNRU:RegionDBS", nlpar="logBPnd"),
  set_prior("normal(0, 0.1)", coef="CentreNRU:RegionHIP", nlpar="logBPnd"),
  set_prior("normal(0, 0.1)", coef="CentreNRU:RegionINS", nlpar="logBPnd"),
  set_prior("normal(0, 0.1)", coef="CentreNRU:RegionOC",  nlpar="logBPnd"),
  set_prior("normal(0, 0.1)", coef="CentreNRU:RegionTHA", nlpar="logBPnd"),
  set_prior("normal(0, 0.1)", coef="CentreNRU:RegionVSTR",nlpar="logBPnd"),

  set_prior("normal(0, 0.1)", coef="CentreNMS:RegionACC", nlpar="logBPnd"),
  set_prior("normal(0, 0.1)", coef="CentreNMS:RegionAMG", nlpar="logBPnd"),
  set_prior("normal(0, 0.1)", coef="CentreNMS:RegionDBS", nlpar="logBPnd"),
  set_prior("normal(0, 0.1)", coef="CentreNMS:RegionHIP", nlpar="logBPnd"),
  set_prior("normal(0, 0.1)", coef="CentreNMS:RegionINS", nlpar="logBPnd"),
  set_prior("normal(0, 0.1)", coef="CentreNMS:RegionOC",  nlpar="logBPnd"),
  set_prior("normal(0, 0.1)", coef="CentreNMS:RegionTHA", nlpar="logBPnd"),
  set_prior("normal(0, 0.1)", coef="CentreNMS:RegionVSTR",nlpar="logBPnd"),

  # Age and clinical covariates (global)
  set_prior("normal(0, 0.1 )", coef="Age_dec_c", nlpar="logBPnd"),
  set_prior("normal(0, 0.1 )", coef="Age_dec_c", nlpar="logk2prime"),
  set_prior("normal(0, 0.2)",  coef="DiagnosisMDD",     nlpar="logBPnd"),
  set_prior("normal(0, 0.2)",  coef="TreatmentPlacebo", nlpar="logBPnd"),
  set_prior("normal(0, 0.2)",  coef="TreatmentKetamine",nlpar="logBPnd"),
  set_prior("normal(0, 0.2)",  coef="TreatmentECT",     nlpar="logBPnd"),
  set_prior("normal(0, 0.2)",  coef="delta_Symptoms",   nlpar="logBPnd"),

  # Region-varying slopes on logBPnd
  set_prior("normal(0, 0.1)",  coef="Age_dec_c",        nlpar="logBPnd",
            group="Region", class="sd"),
  set_prior("normal(0, 0.05)", coef="DiagnosisMDD",     nlpar="logBPnd",
            group="Region", class="sd"),
  set_prior("normal(0, 0.05)", coef="TreatmentKetamine",nlpar="logBPnd",
            group="Region", class="sd"),
  set_prior("normal(0, 0.05)", coef="TreatmentECT",     nlpar="logBPnd",
            group="Region", class="sd"),

  # Heteroscedastic residual model
  set_prior("normal(-0.5, 1)", dpar = "sigma"),
  set_prior("normal(0, 0.3)",  dpar = "sigma", class="sd", group="PET"),
  set_prior("normal(0, 0.1)",  dpar = "sigma", class="sd", group="Region:Centre"),
  set_prior("normal(0, 0.3)",  coef="logRegSize_c", dpar = "sigma", class="b"),
  set_prior("normal(0, 0.1)",  coef="logRegSize_c", dpar = "sigma",
            group="Centre", class="sd"),
  set_prior("normal(1, 0.3)",  coef="InjRad_logc", dpar = "sigma", class="b"),
  set_prior("normal(0, 0.5)",  coef="dur_logc",    dpar = "sigma", class="b"),
  set_prior("student_t(3, 0, 4)", coef="st_tac:CentreKI_1",  dpar = "sigma", class="b"),
  set_prior("student_t(3, 0, 4)", coef="st_tac:CentreNMS_1", dpar = "sigma", class="b"),
  set_prior("student_t(3, 0, 4)", coef="st_tac:CentreNRU_1", dpar = "sigma", class="b"),
  set_prior("student_t(3, 0, 2.5)", dpar = "sigma", class="sds")
)
```

6.2 Supplementary Materials S2: Random Heterogeneity

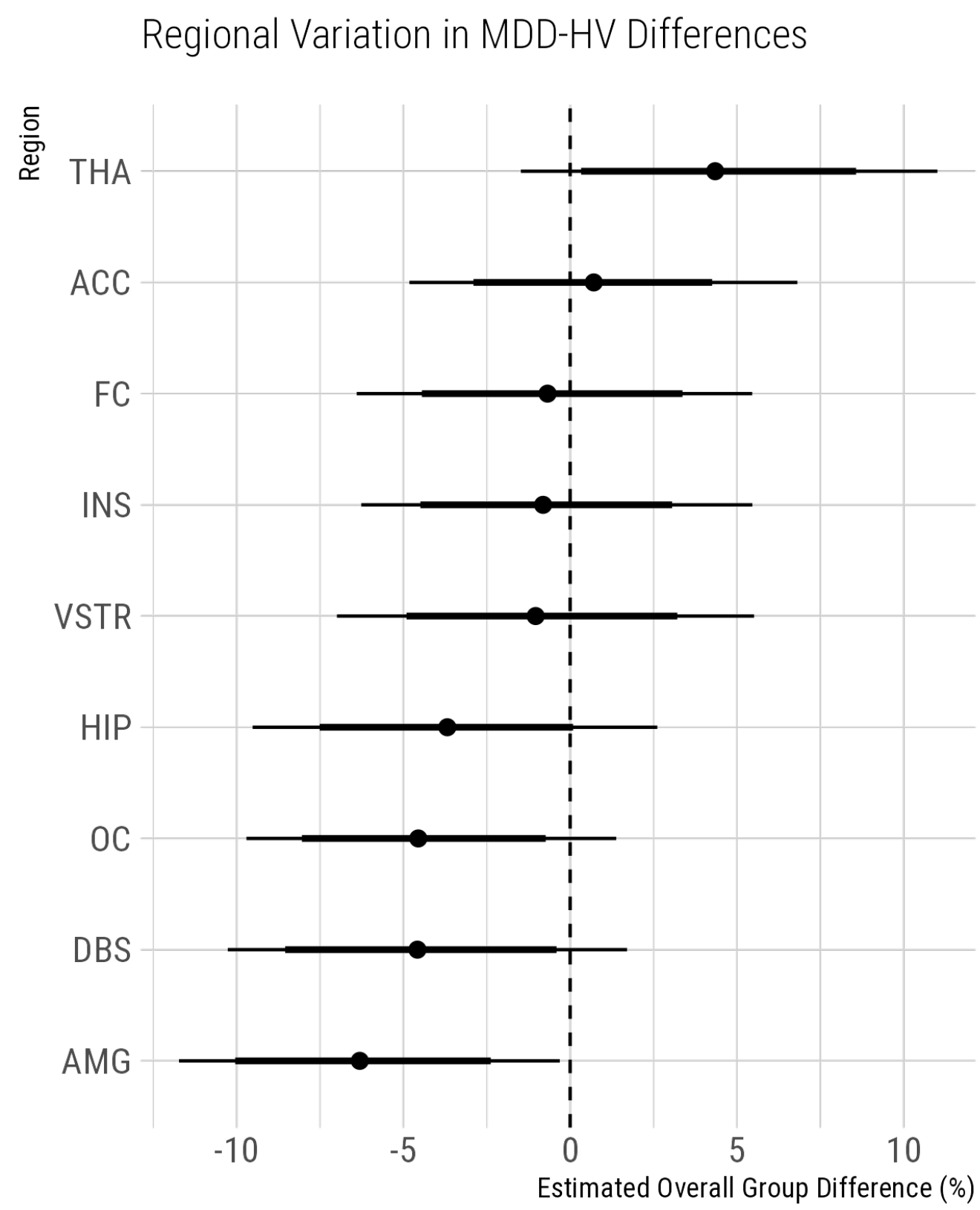

Figure S1. After combining the global fixed effect with the random regional deviations, the combined regional deviations representing differences between MDD and healthy volunteers are shown above for each region. Thicker lines represent the 80% credible intervals while the thinner lines represent the 95% credible intervals. Regional abbreviations are as follows: ACC is anterior cingulate cortex, AMG is amygdala, DBS is dorsal brain stem, FC is frontal cortex, HIP is hippocampus, INS is insula, OC is occipital cortex, THA is thalamus, and VSTR is ventral striatum.

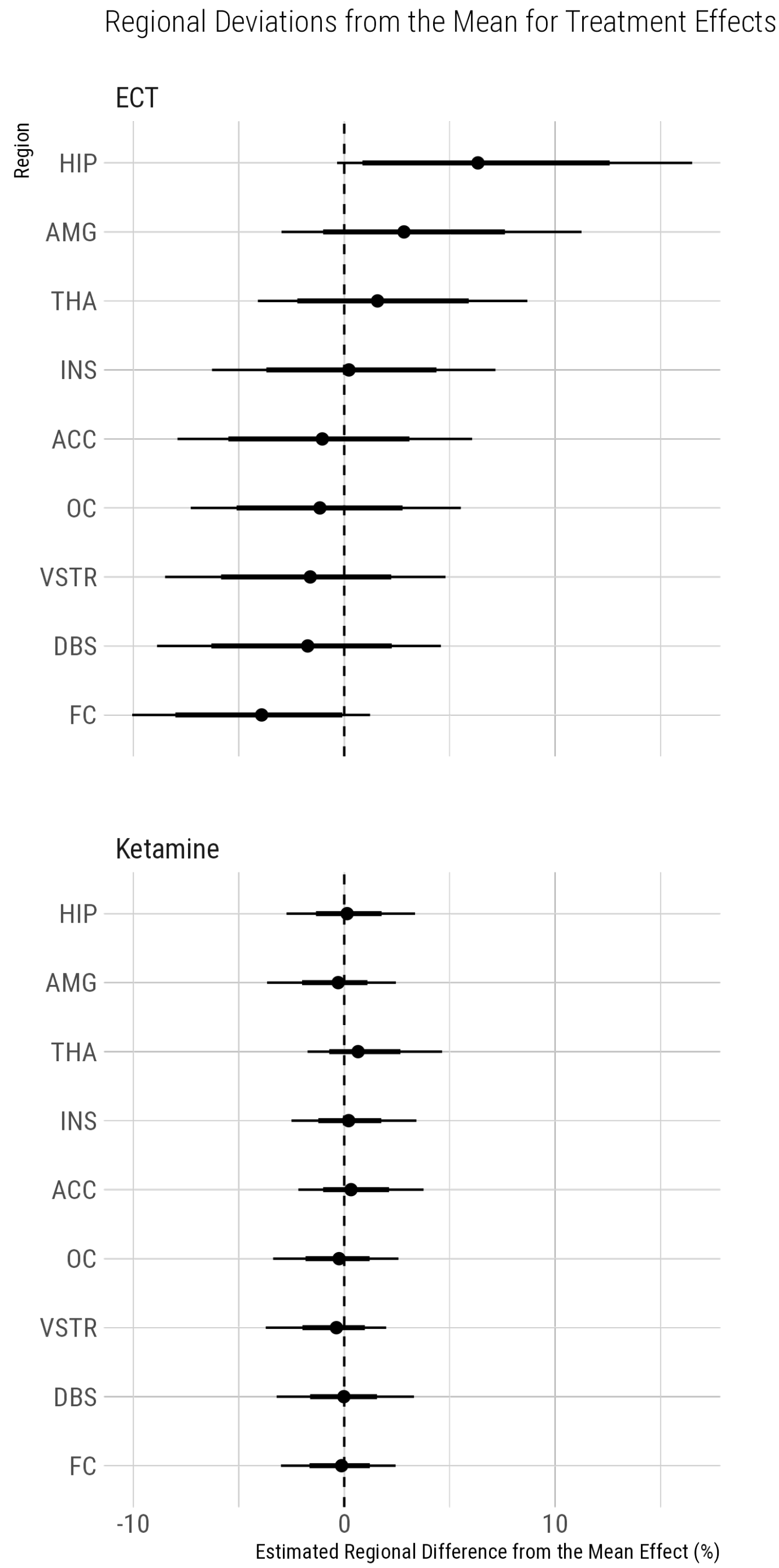

Figure S2. Comparing the random regional deviations from the overall mean effects at 0, the regional hetereogeneity of treatment effects of ECT and ketamine are shown above. Thicker lines represent the 80% credible intervals while the thinner lines represent the 95% credible intervals. Regional abbreviations are as follows: ACC is anterior cingulate cortex, AMG is amygdala, DBS is dorsal brain stem, FC is frontal cortex, HIP is hippocampus, INS is insula, OC is occipital cortex, THA is thalamus, and VSTR is ventral striatum.

6.3 Supplementary Materials S3: Comparison with Conventional Methods and Benefits of Data Pooling for ECT effects

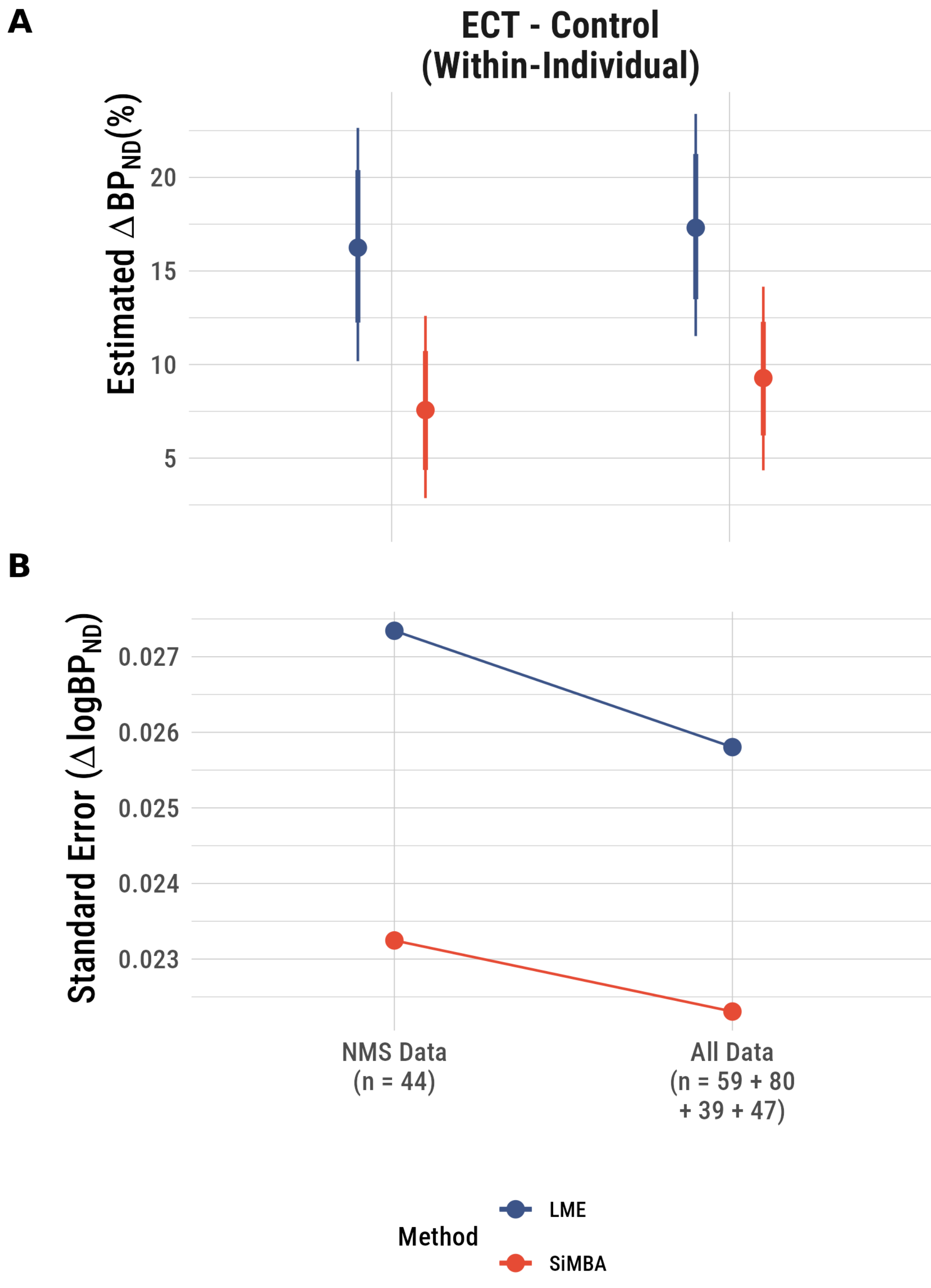

Figure S3. Estimated changes in BP_{ND} following ketamine treatment: evaluation of improvements to inferences using different analysis methods and the effects of including additional data from participants who did not undergo treatment. Blue represents conventional quantification followed by linear mixed effects (LME) analysis, while red represents SiMBA. In A, the difference estimates are shown with thick and thin intervals representing 80% and 95% uncertainty intervals respectively, credible intervals in red and confidence intervals in blue. In B, the SE of the estimates is displayed. In all cases, n represents the number of PET measurements included in the analysis.
